# Shotgun approaches to gait analysis: insights & limitations

**DOI:** 10.1186/1743-0003-11-120

**Published:** 2014-08-12

**Authors:** Ronald G Kaptein, Daphne Wezenberg, Trienke IJmker, Han Houdijk, Peter J Beek, Claudine JC Lamoth, Andreas Daffertshofer

**Affiliations:** MOVE Research Institute Amsterdam, Faculty of Human Movement Sciences, VU University Amsterdam, Amsterdam, The Netherlands; Heliomare R&D, Wijk aan Zee, The Netherlands; University Medical Centre Groningen, Center of Human Movements Sciences Groningen, University of Groningen, Groningen, The Netherlands; School of Sport and Education, Brunel University, London, UK

**Keywords:** Gait, Coordination dynamics, Data analysis

## Abstract

**Background:**

Identifying features for gait classification is a formidable problem. The number of candidate measures is legion. This calls for proper, objective criteria when ranking their relevance.

**Methods:**

Following a shotgun approach we determined a plenitude of kinematic and physiological gait measures and ranked their relevance using conventional analysis of variance (ANOVA) supplemented by logistic and partial least squares (PLS) regressions. We illustrated this approach using data from two studies involving stroke patients, amputees, and healthy controls.

**Results:**

Only a handful of measures turned out significant in the ANOVAs. The logistic regressions, by contrast, revealed various measures that clearly discriminated between experimental groups and conditions. The PLS regression also identified several discriminating measures, but they did not always agree with those of the logistic regression.

**Discussion & conclusion:**

Extracting a measure’s classification capacity cannot solely rely on its statistical validity but typically requires proper post-hoc analysis. However, choosing the latter inevitably introduces some arbitrariness, which may affect outcome in general. We hence advocate the use of generic expert systems, possibly based on machine-learning.

## Background

The assessment of movement is developing rapidly as a result of recent advances in data acquisition. Multiple signals can be readily recorded for considerable time spans. Parallel progress in data analysis allows for a combined application of more conventional, multivariate statistics like principal or independent components and stability-related measures, e.g., standard deviation of relative phase, Lyapunov exponents, and Floquet multipliers [1–5]. In the study of human gait, this led to many important findings regarding, e.g., the coordinative stability and adaptability of walking in relation to speed, curved walking, age and various pathologies including stroke, Parkinson’s disease, cerebral palsy, pregnancy-related pelvic pain, amputations and low back pain [6–16]. Collectively, these studies have demonstrated the expediency of various ‘novel’ measures in characterizing gait dynamics and their surplus value relative to ‘traditional’ kinematic measures pertaining to more isolated features of walking.

Despite the progress in data acquisition and analysis, several limitations have come to the fore. For instance, there is culminating evidence that stride fluctuations in young, healthy humans are characterized by a power law-like behavior (i.e., long-term correlations) that tends to vanish with age [17] and pathology (e.g., [18, 19]). Although important as a general observation, the underlying measure (scaling exponent) proved not sufficiently specific to differentiate between gait-related pathologies. Likewise, the stability-related measures have been instrumental in revealing task- and patient-specific changes in gait dynamics, but, as it stands, it is not evident how these measures relate to gait stability in a biomechanical sense, to proneness to falls, and to the various variability measures (e.g., standard deviation and coefficient of variation of stride duration). These limitations can be seen as derivatives from what we view as a potential problem of current gait analysis, namely that often measures and qualifiers are used that are selected a priori without thorough (theoretical) considerations. When this is the case, a more generic, unbiased method to determine a measure’s relevance may be preferred.

We discuss this by following what might be considered a more objective approach: browse through a large set of possible measures and assess their relevance for discriminating gait patterns by evaluating them according to their information content. This approach is typically applied in order to pinpoint measures that are most defining in characterizing different types of gait. Such a task is laborious and time-consuming but can be left to the computer when employing proper statistical evaluation. Statistics has the potential to provide (more) objective criteria for feature selection. This is expected particularly useful in more exploratory studies where, as said, in-depth theoretical underpinnings are lacking.

Our main goal is to gain insight into the feasibility of such a *shotgun* approach for movement assessment. That is, our interest here is not in the specifics of experimental designs and outcomes but in assembling a plenitude of measures and evaluating their capacity for classifying gait. We illustrate this by using data of two previous studies, which provided profound insight into altered gait patterns in amputees and in stroke patients. We ‘blindly’ created a large set of candidate outcome measures and tested them with different statistical approaches. To anticipate, defining the relevance of measures and, by this, reducing the set of measures seems feasible on first sight. Not unexpected, however, results differed between studies and between statistical approaches. Such discrepancies already indicate that also shotgun approaches are confronted by challenges due to the inevitably arbitrary choices regarding variable selection and subsequent statistical evaluation.

## Methods

To demonstrate the shotgun approach we used data from two experimental studies, referred to as study A and B, that involved stroke patients and amputees walking on a treadmill, respectively. In brief, the aim of study A was to investigate the effect of balance support on gait parameters in patients with stroke. Study B addressed the effect of gait speed and of a cognitive dual task on gait of persons with an amputation compared to healthy controls. Primary outcomes of studies A and B have been partly published in [20] and [21], respectively. Study A involved only a single population under three conditions calling for initial statistical assessments in terms of a one-way ANOVA, whereas study B involved two populations implying an initial two-way ANOVA with condition as the within factor and | for study B | group as the between factor. As said, these data merely serve to show benefits and pitfalls of shotgun approaches. For this reason we only provide a very concise outline of the experimental design and data acquisition. More details can be found in [20] and [21].

### Study A - stroke patients

Eighteen patients with stroke participated in study A. Participants walked on a treadmill for five minutes in three different conditions. In the first condition they were not allowed to hold the handrail for support and walked at their corresponding preferred speed. In the second condition participants used the rail for support and walked at their, typically altered, preferred speed. In the third condition, participants still held onto the rail but walking speed was set to the preferred speed of the unsupported condition [20].

Vertical ground reaction forces were obtained using an instrumented treadmill equipped with a force plate. From the ground reaction forces we determined the center-of-pressure (COP) trajectories, *COP*_*x*_ and *COP*_*y*_ (mediolateral [ML] and anterior-posterior [AP] direction, respectively). Trunk accelerations *a*_*x*_, *a*_*y*_, and *a*_*z*_ (ML-, AP-, and vertical direction, respectively) were measured using a tri-axial accelerometer that was mounted near the level of the third lumbar spine segment. In addition, respiration was assessed breath-by-breath using a pulmonary gas exchange system which measured ventilation rate (), oxygen uptake (), carbon dioxide production (), respiratory exchange ratio (*RER*), metabolic costs (*C*_met_) and heart rate (*HR*). *C*_met_ was computed via the oxygen uptake  normalized by bodyweight and walking speed [22]. The data of four participants had to be discarded because of technical problems leaving 14 sets for subsequent analysis.

### Study B - amputees

Study B involved in total 46 participants: 26 with a lower-limb amputation and 21 abled-bodied controls. Of the former group, 16 had a transtibial and 10 a transfemoral amputation [21]. Participants walked on an instrumented treadmill at a comfortable walking speed for four minutes in two different conditions. The first one consisted solely of walking, whereas in the second condition participants performed while walking a cognitive task inducing the Stroop effect: Participants saw a color name printed in color while listening to a spoken color name which either matched or not matched the color of the printed word. Whenever spoken and depicted color disagreed, participants had to press a button. This task had previously been shown to elicit changes in postural control in lower limb amputees during quiet stance [23].

Similar to study A a treadmill with built-in force plate served to measure vertical ground reaction forces during walking. Off-line computation yielded *COP*_*x*_ and *COP*_*y*_. Participants also wore a tri-axial accelerometer mounted near the level of the third lumbar spine segment. The corresponding *a*_*x*_-, *a*_*y*_-, and *a*_*z*_-signals were sampled at a rate of 100 Hz. Oxygen uptake was assessed breath-by-breath using open-circuit respirometry providing , , , *RER*, *C*_met_, *BF*, and *HR*. Furthermore, bilateral surface EMGs were recorded from the tibialis anterior (TA), gastrocnemius (G), vastus medialis (VM), semitendinosus (S), and the tensor fascia latae (TFL), from which the envelopes were estimated (after high-pass filtering at 140 Hz, followed by full-wave rectification using the Hilbert transform, and low-pass filtering at 2 Hz) [24, 25]. Data of several participants had to be discarded because of acquisition problems leaving sets of 19 controls and 19 persons with amputation for analysis.

### Data analysis

We first determined step events based on a fixed moment in the step cycle. An initial estimate for these points in time was obtained using a peak-detection based on the time series of the ground reaction force’s vertical component [26, 27]. The resulting moments were optimized using an iterative template-matching algorithm for the *COP*_*x*_- and *COP*_*y*_-signals which proceeded as follows:

-  select an epoch around every step moment (we used a temporal window of 40% of the mean stride duration, which was estimated via the dominant frequency in the spectral distribution);
-  align the selected epochs and average over step moments – this averaging yields a template;
-  determine per event the time lag at which the serial-lag cross-covariance between epoch and template is maximal and shift the event by that time lag;
-  repeat the entire procedure until convergence.

**Figure 1:**
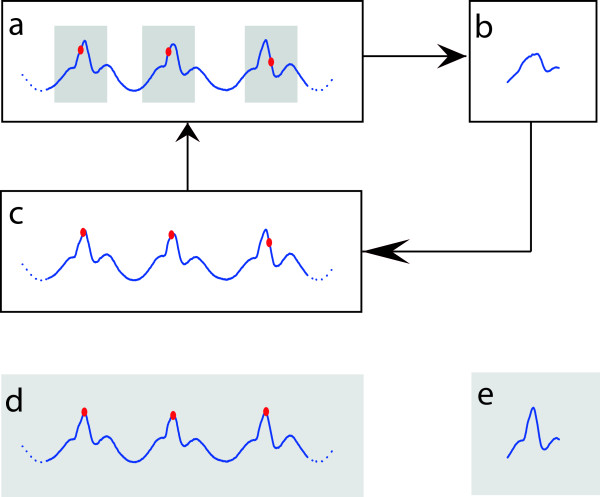
**Illustration of template matching procedure.** The algorithm starts with an initial estimate of events in a signal (panel **a**). Around each event an epoch (window) is defined (grey shading in panel a), and the epochs are averaged to define a template (panel **b**). This template is compared with every epoch using the corresponding cross-correlation function. In the next step, events are shifted by the time lag of maximal cross-correlation (panel **c**). These events then serve as a new initial estimate (panel a). This procedure is repeated until convergence. Panels **d** &**e** show the final events and template.

This procedure is illustrated in Figure 1. The resulting events correspond to a fixed point in the step cycle, which does not necessarily correspond to a distinguishable physical event such as toe off or heel contact. Note, however, that we defined the template such that events where located around the template’s maximum value by which events approximately agreed with moment of heel contact. For study B we applied the same procedure not only to the COP-data but also to the individual EMG-signals so as to obtain proper EMG-moments (∼ EMG-onset) and templates (wave-forms) irrespective of electro-mechanical delays; there we used the optimized force plate events as initial values.

With the so-defined COP-events we estimated mean step width [28], mean stride length, mean stride duration, and their respective coefficients of variation (CV). For both force plate and EMG signals we further derived the mean value and variance of the template over time representing the average mean COP position over the aforementioned temporal window (for the EMG the average rectified value) as well as its mean deviation. Moreover, we determined the mean variance over events averaged over time as measure of the template’s consistency, i.e. the ‘regularity’ of walking and muscle activation. That is, for each data point of the template we calculated the variance using the corresponding points in all epochs, and all these variances were averaged. Regularity of signals wasalso assessed in terms of the corresponding sample entropy [29, 30] following the approach of Lake and co-workers [31] (parameter values: *m*=3 and *r* varying from 0.01 to 0.07). We computed the sample entropy of (Hilbert-)amplitude and (Hilbert-)phase of the force plate, EMG and accelerometer signal.

To address other complexity-related features we analyzed the signals’ temporal correlation structure using detrended fluctuation analysis (DFA) [32, 33]. DFA yields a parameter *α* (scaling index or self-similarity parameter) that equals the so-called Hurst exponent [18] under the assumption that the generating process represents fractal Gaussian noise: A fully random signal (white noise) corresponds to *α*=0.5; signals containing anti-persistent correlations have *α*<0.5, and persistent correlations imply *α*>0.5. Finally, we determined the maximum Lyapunov exponents as measure for (local, dynamic) gait stability [34–37]. We employed Rosenstein and co-workers’ algorithm with embedding dimension *M*=7 (estimated via the number of false nearest neighbors) and *τ*=20 samples embedding delay (estimated via the first minimum of the average mutual information) [38]. Maximum Lyapunov exponents were determined for the EMG and acceleration signals as well as for their principal components. The latter were determined through conventional principal component analysis (PCA) [39], from which we also stored the corresponding eigenvalue spectrum for subsequent statistical assessment [36] (i.e. three and five eigenvalues for accelerometer and EMG signals, respectively).

### Measures

The following measures entered our statistical assessments – we sorted measures by recording modality; where numbers in between parentheses indicate for how many dimensions, channels, or muscles a measure was determined (e.g., *x*, *y*, and *z* for accelerometer data and *COP*_*x*_, *COP*_*y*_, and *F*_*z*_ for force plate data).

*Ground reaction force*: mean and CV of step width, stride length, and stride duration; DFA- *α* of stride duration, mean and CV of event position (3), DFA- *α* of event position (3), template mean (3), mean variance over events (3), sample entropy (3), standard error of sample entropy (3), sample entropy of phase (3), standard error of sample entropy of phase (3).

*Accelerometry*: max. Lyapunov exponent (3), sample entropy (3), standard error of sample entropy (3), PCA eigenvalues (3), max. Lyapunov exponent of principal components (3).

*Metabolism*: mean and CV of , , , *RER*, *BF*, *C*_met_, and *HR*; no breathing-frequency data were available for stroke patients, i.e. there we used only twelve metabolic measures.

*EMG*: median frequency (5), template mean (5), mean variance over events (5), sample entropy (5), standard error of sample entropy (5), max. Lyapunov exponent (5), PCA eigenvalues (5), max. Lyapunov exponents of PCA components (5), sample entropy of phase (5), standard error of sample entropy of phase (5).

The total number of distinct (though possibly correlated) measures was 61 for study A and 113 for study B.

### Hypothesis testing

As a first step, we tested main and interaction effects for all measures considered. We conducted separate repeated measures ANOVAs with *condition* as the within factor and–for study B–*group* as the between factor. To facilitate comparison of study A with study B we also performed supplementary analyses for study B by determining first the relative difference (i.e., the difference between the measures of the two conditions divided by their mean) that were entered into a one-way ANOVA as in study A. Because the aim of our shotgun approach was to “identify relevant measures in (changes of) gait patterns” we preferred individual ANOVAs over multivariate approaches (e.g., MANOVA, PCA) [40]. Note that the very large number of measures typically incorporated in shotgun approaches renders MANOVAs in general less feasible.

**Figure 2:**
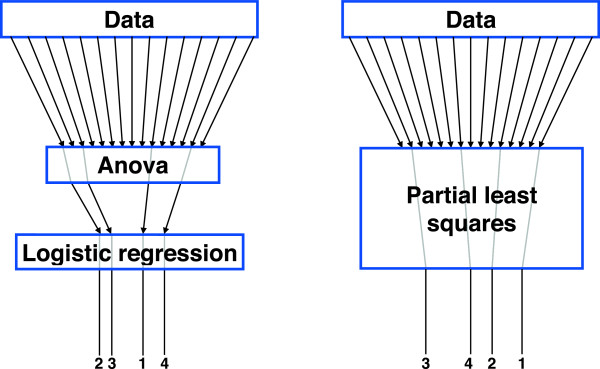
**Schematic overview of the statistical assessment.** The left panel depicts that in the first alternative all the measures are first analyzed using a conventional ANOVA. This extracts the subset of most significant measures, which is subsequently assessed using a multinomial logistic regression. This regression yields a certain ordering of measures-of-interest. The right panel shows the second alternative, in which the selection plus ordering is realized via a PLS regression; see the text for more details.

A Bonferroni correction was applied to correct for type I errors due to the large number of ANOVAs. Results were considered significant if the corresponding *p*-value did not exceed 0.05. Given the large number of incorporated measures, a Bonferroni correction like every other correction for multiple comparison comes with problems. This will be discussed below in detail. On account of these problems we supplemented the post-hoc analysis by two alternatives, a multinomial logistic regression and a partial least squares regression. All approaches are sketched in Figure 2.

### Multinomial logistic regression

In order to determine which of the measures constitute the best predictors for an experimental factor (group or condition), we used nominal multinomial logistic regression models. In a nutshell, such models fit the log-probability that a nominal factor falls in a certain category, expressed as a fraction of the probability that it falls in another category, i.e. . In this expression *π*_*j*_ denotes the probability that the factor of interest is in category *j*, *k* represents the reference category, *x*_*i*_ are the values of the *i*^th^ measurement, *β*_*ij*_ are the regression coefficients, and *n* is the number of included measures. Since we incorporated three conditions in study A and three groups in study B, the regression initially returned two vectors of *β*-coefficients, namely *β*_13_ and *β*_23_ (number 3 is the reference group). However, the third coefficient *β*_12_ simply equals *β*_13_−*β*_23_. To estimate the overall importance of a measure, we finally determined the *β*-norm (i.e. ). Here it is important to realize that we considered more measures than observations. Therefore the number of measures in the regression had to be constrained. We selected measures using the corresponding *p*-values resulting from the individual ANOVAs; see Figure 2, left panel. We used either only the measures with *p*<0.05/*N* or sorted all measures by *p*-value in descending order and considered the first seven values only^a^. All measures were normalized to *z*-scores before entering the regression. For study B we used the relative difference between the measures of the two conditions as input for the regression.

The overall performance of the regression was quantified in terms of the correct rate. For this, we first computed the *β*-coefficients. Next, we determined the predicted probabilities for each category and classified the observation according to the highest probability. Then, the correct rate was given by the fraction of observations classified properly. Note that it would have been more appropriate to divide the observations into a training and a test set, but the number of observations was not large enough to do so.

### Partial least squares regression

Partial least squares (PLS) regression deals with correlated measures by decomposing in- and output into factor scores which are linearly independent. PLS is similar to principal component analysis (PCA) but PCA relies on the covariance between input variables, whereas PLS capitalizes on the covariance between the input and response variables. In brief, PLS decomposes the input ***X*** (*n* observations ×*m* measures) and the output ***Y*** (*n* observations ×*k* categories) into ***X***=***TP***^*t*^+***δ*** and ***Y***=***UQ***^*t*^+***ε***, respectively. ***T*** and ***U*** are the score matrices (both *n* × *l*), ***P*** and ***Q*** are the loading matrices (*n*×*m* and *k* × *l*, respectively), ***δ*** and ***ε*** are residuals, and *l* denotes the number of components. Presuming a linear relation between ***T*** and ***U***, these equations can be combined in the form ***Y***≈***Xβ***, which is the searched-after linear model [41].

Since we included nominal variables (condition and group), we used an indicator matrix as the response variable [41]. As in conventional PCA, we here limited the number of new components used in the regression, i.e. *l* ≪ *m*. In particular, we used seven components in the PLS regression in agreement with the aforementioned multinomial logistic regression^1^; cf. Results. Note that testing for significance of the PLS regression is possible but not straightforward [42]. For the sake of brevity we here decided not to add such a test. Quantification of performance was realized in the same way as in the multinomial logistic regression, i.e. using the correct rate; see Figure 2.

## Results

### Hypothesis testing

Table 1 summarizes the ANOVA results for study A (stroke data). Only three of the 61 measures revealed significant differences between conditions, all three being metabolic measures. Using the unaffected side for the step-related measures did not alter results.

**Table 1:** **Measures from the participants with stroke that show a significant condition effect**

Modality	Measure	Condition
Metabolism	Mean	0.00007 (0.72)
	Mean	0.00005 (0.73)
	Mean	0.0002 (0.66)

The effect size (partial *η* ^2^) is indicated between brackets. The significance level after the Bonferroni correction was 0.0009.

The repeated measures two-way ANOVA results for study B (amputation data) are given in Table 2. Two measures displayed a significant main effect of condition and 14 measures showed a main effect of group. Of these 14 group effects, two were only significant for the affected leg. No significant interaction effects were found. The result of the one-way ANOVA for study B using the relative outcome measures also revealed no significant effects.

**Table 2:** **Measures from the participants with an amputation that showed a significant effect**

Modality	Measure	Condition	Group	Interaction
Force plate	Mean step width	-	0.00004 (0.95)	-
	Mean stride duration	-	0.0004 (0.94)	-
	CV of stride duration	-	0.0002 (0.66) [1]	-
	Template mean (x)	-	0.0003 (0.34) [1]	-
	Mean variance events (z)	-	0.0004 (0.77)	-
	SE of sample entropy (y)	-	0.00001 (0.94)	-
	SE of sample entropy (z)	-	0.0002 (0.75)	-
	Sample entropy of phase (z)	-	0.0004 (0.98)	-
	SE of sample entropy of phase (y)	-	0.00001 (0.97)	-
EMG	Sample entropy (S)	-	0.0001 (0.87)	-
	Lyapunov exponent (G)	-	0.0004 (0.50)	-
Accelerometer	Sample entropy (x)	-	0.00003 (0.69)	-
	PCA eigenvalues (1)	-	0.0002 (0.48)	-
Metabolism	Mean	0.0002 (0.32)	-	-
	Mean *BF*	0.0002 (0.32)	-	-
	Mean *C*_met_	-	< 0.00001 (0.96)	-

The effect size (partial *η* ^2^) is indicated between brackets. If a leg specific measure such as stride length was only significant for one leg, this is indicated between square brackets; 1 stands for the affected side, 2 for the unaffected side (see text). If nothing is indicated, both legs showed a significant effect, and the reported *p* value is for the affected side. Note that the significance level is 0.0005 after the Bonferroni correction.

### Multinomial logistic regression

The multinomial logistic regression for study A resulted in a correct rate of 79% when using the seven measures with the smallest *p*-values (this reduced to 40% when restricting to only the three significant measures). The corresponding *β*-coefficients are summarized in Table 3, which also contains the *p*-values from the one-way ANOVA. The three measures with the largest *β*-norm were the mean ventilation rate , the Lyapunov exponent of the accelerometer *y*-signal (AP-direction), and the mean metabolic rate *C*_met_. Note the difference with the ANOVA results in Table 1. Both the mean  and the DFA- *α* of stride duration were significant for *β*_13_ and for *β*_23_.

**Table 3:** ***β*** **-coefficients of the logistic regression for study A, using the 7 measures with the highest** ***p*** **-value (2nd column)**

Measure	***p***	***β***_13_	***β***_23_	***β***_12_	|| ***β***||
Metabolism mean	0.00007	-5.2*	-4.1*	-1.1	6.7
Accelerometer lyapunov exponent (y)	0.01	-2.0	-4.6*	2.6	5.7
Metabolism mean *C*_met_	0.02	3.5	3.4*	0.1	4.8
Metabolism mean	0.0002	2.5	-1.3	3.8	4.7
Metabolism mean	0.00005	0.5	3.4	-2.9	4.5
Accelerometer lyapunov of PC (1)	0.02	-2.3	0.5	-2.8	3.6
Force plate DFA- *α* of stride duration	0.02	-2.6*	-1.3*	-1.3	3.2

The significance level for these *p*-values is 0.0009 after the Bonferroni correction. Significant coefficients (*p*<0.05) are indicated with an *. For *β*_12_ and ||*β*|| no significance could be indicated because these were calculated after the regression. Note that in the *β*-coefficients, 1 indicates the supported preferred speed condition, 2 indicates the supported enforced speed condition and 3 indicates the unsupported preferred speed condition.

For study B, we performed the regression using the results from the one-way ANOVA that used the relative difference between the outcomes of the two conditions as input. When again using the seven measures with largest *p*-values, we found a correct rate of 77%. The regression coefficients are listed in Table 4. The CV of the AP event position of the force plate data (*COP*_*y*_) yielded the largest *β*-norm.

**Table 4:** ***β*** **-coefficients of the logistic regression for study B, using the relative difference between non-Stroop and Stroop results as measure**

Measure	***p***_1_	***p***_2_	***β***_13_	***β***_23_	***β***_12_	|| ***β***||
Force plate SE of sample entropy (y)	0.02	0.03	-2.8	-0.6	-2.1	3.5
EMG sample entropy (TA)	0.006	0.005	0.3	-1.8	2.1	2.8
Force plate DFA- *α* of stride duration	0.09	0.1	1.2*	-0.7	1.9	2.4
EMG SE of sample entropy of phase (VM)	0.05	0.7	-1.2	0.6	-1.8	2.3
Metabolism mean (HR)	0.07	0.08	-1.5	-0.2	-1.3	2.0
EMG PCA eigenvalues (5)	0.07	0.02	-0.8	-1.3	0.5	1.6
Force plate mean template (z)	0.07	0.4	0.7	0.8	-0.1	1.1

The 7 measures with the highest *p*-value were included. The second column (*p*_1_) shows the *p*-value for the one-way ANOVA using the alternative measure, the third column (*p*_2_) shows the *p*-values for the interaction effect of the two-way ANOVA. Note that in both cases, the significance level was 0.0004 after the Bonferroni correction. Significant coefficients (*p*<0.05) are indicated with an *. For *β*_12_ and ||*β*|| no significance could be indicated because these were calculated after the regression. Note that for the *β*-coefficients, 1 indicates the transtibial amputee group, 2 the transfemoral group and 3 the control group.

### PLS regression

The PLS regression for study A resulted in a correct rate of 88% (recall that also here we included seven components). The measure with the highest *β*-norm turned out to be the DFA- *α* of stride duration. The results of the regression are given in Table 5.

**Table 5:** **Regression coefficients of the PLS regression for study A**

Measure	***β***_1_	***β***_2_	***β***_3_	|| ***β***||
Force plate DFA- *α* of stride duration	0.41	-0.25	-0.16	0.51
Force plate DFA- *α* of event position (z)	-0.14	-0.22	0.36	0.44
Force plate mean template (z)	0.14	0.15	-0.29	0.36
Force plate DFA- *α* of event position (y)	0.25	-0.25	-0.01	0.35
Metabolism cv of RER	-0.11	-0.17	0.28	0.35
Force plate SE of sample entropy (z)	0.07	-0.24	0.17	0.30
Force plate mean event position (z)	0.01	-0.21	0.19	0.29

The number of components included in the regression was set to 7. *β*_1_ indicates the supported preferred speed condition, *β*_2_ the supported enforced speed condition and *β*_3_ the unsupported preferred speed condition.

For study B, the PLS regression using the relative difference yielded a correct rate of 100%. The two measures with the highest *β* norm are the DFA- *α* of stride duration and the standard error of the sample entropy of the *COP*_*y*_-signal (AP direction). All *β*-coefficients of the regression can be found in Table 6.

**Table 6:** **Regression coefficients of the PLS regression for study B**

Measure	***β***_1_	***β***_2_	***β***_3_	|| ***β***||
Force plate SE of sample entropy (y)	0.21	-0.21	-0.02	0.29
Force plate DFA- *α* of stride duration	-0.17	0.23	-0.05	0.29
EMG SE of sample entropy (VM)	-0.16	0.22	-0.06	0.28
Force plate sample entropy (y)	0.19	-0.09	-0.14	0.25
Accelerometer sample entropy (z)	0.17	-0.05	-0.15	0.24
EMG PCA eigenvalues (5)	0.16	-0.07	-0.11	0.21
EMG SE of sample entropy of phase (VM)	0.01	-0.14	0.14	0.20
EMG sample entropy (TA)	0.13	-0.02	-0.14	0.19
EMG PCA eigenvalues (1)	-0.11	-0.01	0.15	0.19
EMG SE of sample entropy of phase (S)	0.12	-0.14	0.02	0.19

The number of components included in the regression was set to 7. *β*_1_ indicates the control group, *β*_2_ the transtibial amputee group and *β*_3_ transfemoral group.

## Discussion

Shotgun approaches for analyzing multivariate gait signals can provide important insights into gait classification but they are also confronted with considerable challenges. We used data of two experimental studies to illustrate these challenges. Study A had a one-way design as the one patient group was tested in three conditions: walking unsupported at their preferred walking speed, supported at their preferred speed, and walking supported at their preferred speed of the unsupported condition. Study B incorporated three groups, two types of lower-limb amputees and a control group, that were assessed in two conditions (i.e. a two-way design). In both cases we determined large sets of outcome measures that underwent different statistical assessments; see Figure 2 for a schematic overview.

Conventional ANOVAs returned significant main effects for study A and B, in the absence of significant interaction effects for study B. In study A, three metabolic measures were found to be significant (c.f., [20]), in study B, stride length and metabolic costs revealed group effects (e.g., [43–45]). The absence of more main effects that could be expected based on the established literature might be explained by the correction for multiple comparisons. The Bonferroni correction that we used is the most common one, but it is also quite conservative. Of course, we could have chosen alternatives like the Šidák correction, or even could have followed an entirely different route, like the Benjamini-Hochberg procedure [46]. However, this would not have helped to overcome the fundamental problem: A shotgun approach may involve such an arbitrarily large set of measures that conventional statistical assessments (e.g., ANOVAs) simply become unfeasible; just adding measures will inevitably suppress significance and, on top of that, measures may be correlated, which may hamper analysis too.

We followed alternative routes, albeit equally arbitrary, and employed a multinomial logistic as well as a partial least squares regression. For the logistic regression, we used the ANOVA results to limit the number of input measures, while we used all measures in the PLS regression. Both regressions performed well in terms of success rate, which varied from 77% to 100%, indicating that the regression weights are indicative of a measure’s relative classification capacity. The logistic regressions also returned multiple significant coefficients. Notably, the regressions for study B, targeted at examining the interaction effect, performed very well while no interaction effect was found in the ANOVA.

It may be difficult to find clear correlations between many of the chosen measures and complex gait patterns. The ‘insufficiency’ of the conventional ANOVAs and the ‘improved’ performance of the logistic and PLS regression in revealing the measures’ relationships with experimental groups and conditions may be self-evident. However, when comparing the results of the logistic regression with those of the PLS regression one can realize that the measures identified as relevant differ between approaches. For example, the mean ventilation rate  has the highest weight in the logistic regression for study A (Table 3), but it does not appear at all in the results of the PLS regression (Table 5). For study A, only one measure appears in both regressions, namely the DFA- *α* of stride duration. For study B, the results are more similar, with 5 of the 7 measures appearing in both tables. Both statistical post-hoc assessments appear valid, at least from a more mathematical perspective, with the addition that PLS regression accounts for possible correlations between measures. We did expect results to differ but having said that the question remains: How can one decide which measures are appropriate? As the number of measures continues to increase, this question will become more important.

There are several more caveats to mention. For example, for study B we used the relative difference between the measures of the two conditions in the regression analysis. We could have chosen the ratio, the log-difference, or any other measure defining a proper metric. Similarly arbitrary was fixing the threshold to seven when ranking *p*-values in the logistic regression, or in fact choosing *p*-values for ranking instead of, for example, effect sizes. Moreover, the calculation of some of the outcome measures required making other arbitrary choices, such as the temporal window size in the event optimization procedure and the embedding parameters for the Lyapunov exponent, et cetera et cetera.

The logistic and PLS regressions are two selected additional methods to identify relevant measures. Statistics comes with a plethora of assessment methods from which most can be used in various (overlapping) circumstances. Our goal to stay as objective as possible is once more at stake because again we have to make somewhat arbitrary choices. That is, pushing the shotgun approach as far as possible by keeping it largely objective by including as little as possible a priori knowledge turned out to be quite challenging. While the feature definition progressed profoundly, the feature selection awaits to be implemented in a similarly advanced way. Especially in view of ‘big data’, i.e. the massive amount of data being collected to date, an apropriate feature selection is mandatory. One possibility for this would be to supplement the measure extraction by more generic, data-driven expert systems that capitalize on a priori data reduction [39, 47] – though here one simply pushes the selection problem back to the definition of measures. Alternatives are machine-learning techniques for pattern classification that are slowly penetrating gait analysis (e.g., [48–62]). We are currently developing a partly unsupervised learning approach that incorporates the literature-based educated guesses for measure selection. In future work we will compare this to the current study. In fact, all the here discussed signal-processing steps have already been implemented as an open-source toolbox [63].

We advocate to progress data analysis towards machine learning in combination with theory-based (i.e. educated) selection criteria. By the same token, however, we believe that shotgun approaches can be a valid addition when data-mining. As we illustrated in detail how post-hoc assessments may point at relevant measures and unravel interesting effects that may warrant further investigation. As it stands, however, proper insight into measure selection and post-hoc statistics remains mandatory.

## Conclusion

Shotgun approaches can help identifying measures for gait assessment. This is particularly true if measures are incorporated that still lack a theoretical underpinning in the context of gait analysis. In order to extract a specific measure’s classification capacity, however, one cannot solely rely on its statistical validity (e.g., the outcome of an ANOVA). For the current examples we showed that both logistic as well as partial least squares regression produced interesting albeit diverging results. Both post-hoc analyses can be defended from a statistical/mathematical perspective. This difference is seminal for what we believe is a major concern: At some point in the analysis arbitrary choices are inevitable. While the outlined procedure may be useful in many situations, we believe that in order to create a blind approach, more generic methods are required. In this context one can think of machine-learning techniques or even more general expert systems borrowed from the field of artificial intelligence.

## Consent

Written informed consent was obtained from all the participants for the publication of this report.

## Endnote

^a^ Introducing a threshold always implies a degree of arbitrariness. Selecting *p*<0.05 to define significance is as arbitrary as selecting the seven largest *p*-values as done here. In fact we chose that cut-off at will because in the current study it only served to improve legibility, although there is a limit to the number of measures that can be included due to the finite sample size.
